# [Retracted] Downregulated microRNA‑140‑5p expression regulates apoptosis, migration and invasion of lung cancer cells by targeting zinc finger protein 800

**DOI:** 10.3892/ol.2024.14727

**Published:** 2024-10-07

**Authors:** Enqing Zhuo, Changqing Cai, Wenzhe Liu, Kunsong Li, Wenzhen Zhao

Oncol Lett 20: 390, 2020; DOI: 10.3892/ol.2020.12253

Following the publication of this paper, it was drawn to the Editor's attention by a concerned reader that certain of the Transwell cell migration assay data shown in Fig. 2A on p. 5 had already appeared in a couple of previously published articles written by different authors at different research institutes. Owing to the fact that the contentious data in the above article had already been published prior to its submission to *Oncology Letters*, the Editor has decided that this paper should be retracted from the Journal. The authors were asked for an explanation to account for these concerns, but the Editorial Office did not receive a reply. The Editor apologizes to the readership for any inconvenience caused.

